# Was It Chikungunya? Laboratorial and Clinical Investigations of Cases Occurred during a Triple Arboviruses’ Outbreak in Rio de Janeiro, Brazil

**DOI:** 10.3390/pathogens11020245

**Published:** 2022-02-14

**Authors:** Thiara Manuele Alves de Souza, Raquel Curtinhas de Lima, Victor Edgar Fiestas Solórzano, Paulo Vieira Damasco, Luiz José de Souza, Juan Camilo Sanchez-Arcila, Gabriel Macedo Costa Guimarães, Iury Amâncio Paiva, Monique da Rocha Queiroz Lima, Fernanda de Bruycker-Nogueira, Larissa Cristina Teixeira Tomé, Mariana Rosa Inácio Coelho, Sandro Patroca da Silva, Luzia Maria de Oliveira-Pinto, Elzinandes Leal de Azeredo, Flavia Barreto dos Santos

**Affiliations:** 1 Viral Immunology Laboratory, Oswaldo Cruz Institute, Rio de Janeiro 21040-360, Brazil; thiara.biomed@gmail.com (T.M.A.d.S.); raquellima@aluno.fiocruz.br (R.C.d.L.); vicfiso@gmail.com (V.E.F.S.); juancamilos@gmail.com (J.C.S.-A.); gabrielmcguimaraes@gmail.com (G.M.C.G.); iury.iap@gmail.com (I.A.P.); moniquerql@gmail.com (M.d.R.Q.L.); larissatome.biomed@gmail.com (L.C.T.T.); marianarosa99b@gmail.com (M.R.I.C.); lpinto@ioc.fiocruz.br (L.M.d.O.-P.); elzinandes@ioc.fiocruz.br (E.L.d.A.); 2 Rio-Laranjeiras Hospital, Rio de Janeiro 22240-000, Brazil; paulovieiradamasco@gmail.com; 3 Gaffrée Guinle University Hospital, Federal University of the State of Rio de Janeiro, Rio de Janeiro 20270-003, Brazil; 4 Pedro Ernesto University Hospital, University of the State of Rio de Janeiro, Rio de Janeiro 20551-030, Brazil; 5 Plantadores de Cana Hospital, Campos dos Goytacazes, Rio de Janeiro 28025-496, Brazil; luizjosedes@gmail.com; 6 Flavivirus Laboratory, Oswaldo Cruz Institute, Rio de Janeiro 21040-360, Brazil; nandanog@ioc.fiocruz.br; 7 Department of Arbovirology and Haemorrhagic Fevers, Evandro Chagas Institute, Ananindeua 67030-000, Brazil; spatroca@gmail.com

**Keywords:** arboviruses, chikungunya, triple epidemic, differential laboratorial diagnosis, phylogeny, ECSA genotype

## Abstract

The co-circulation of chikungunya virus (CHIKV), dengue virus (DENV) and Zika virus (ZIKV) in Rio de Janeiro (RJ), Brazil, caused a challenging triple epidemic, as they share similar clinical signs and symptoms and geographical distribution. Here, we aimed to investigate the clinical and laboratorial aspects of chikungunya suspected cases assisted in RJ during the 2018 outbreak, focusing on the differential diagnosis with dengue and zika. All suspected cases were submitted to molecular and/or serological differential diagnostic approaches to arboviruses. A total of 242 cases suspected of arbovirus infection were investigated and 73.6% (178/242) were molecular and/or serologically confirmed as chikungunya. In RT-qPCR confirmed cases, cycle threshold (Ct) values ranged from 15.46 to 35.13, with acute cases presenting lower values. Chikungunya cases were mainly in females (64%) and the most frequently affected age group was adults between 46 to 59 years old (27%). Polyarthralgia affected 89% of patients, especially in hands and feet. No dengue virus (DENV) and Zika virus (ZIKV) infections were confirmed by molecular diagnosis, but 9.5% (23/242) had serological evidence of DENV exposure by the detection of specific anti-DENV IgM or NS1, and 42.7% (76/178) of chikungunya positive cases also presented recent DENV exposure reflected by a positive anti-DENV IgM or NS1 result. A significantly higher frequency of arthritis (*p* = 0.023) and limb edema (*p* < 0.001) was found on patients with CHIKV monoinfection compared to dengue patients and patients exposed to both viruses. Lastly, phylogenetic analysis showed that the chikungunya cases were caused by the ECSA genotype. Despite the triple arboviruses’ epidemic in the state of RJ, most patients with fever and arthralgia investigated here were diagnosed as chikungunya cases, and the incidence of CHIKV/DENV co-detection was higher than that reported in other studies.

## 1. Introduction

Chikungunya virus (CHIKV) belongs to the *Togaviridae* family and to the *Alphavirus* genus [1,2], having a spherical and enveloped viral particle that measures approximately 60–70 nm in diameter. The genome consists of a single-stranded positive polarity RNA that measures approximately 11.8 kb in length, encoding four non-structural proteins: NSP1–4, and five structural proteins: C-E3-E2-6K-E1 [1,3,4]. Three different CHIKV genotypes have been described: West African, East-Central-South African (ECSA) and Asian [5,6].

Chikungunya Fever is characterized as an acute febrile illness with sudden onset and can last for up to two weeks, with high fever, usually with maculopapular eruptions on the trunk and extremities, headache, myalgia and intense polyarthralgia, mainly in the distal joints, and edema in the region. The gastrointestinal tract can also be affected, causing nausea, vomiting and diarrhea [7]. The symptoms may last for three months, when the disease is considered to have reached the subacute form, or even years, causing severe, debilitating and persistent arthralgia [8,9].

Since its first description in 1952 in Africa [10,11], CHIKV has caused emerging and reemerging epidemics in several regions of the world. Due to the transmission dynamics, it has preferably reached regions of tropical climate [9,12]. The urban transmission cycle of CHIKV involves vertebrate hosts and hematophagous mosquitoes *Ae. aegypti* and *Ae*. *Albopictus* [13,14,15], with the human being the only host capable of developing the clinical forms of the infection [16,17,18]. Many factors contribute to the spread of CHIKV, such as environmental determinants, presence of susceptible vectors, human behavior and population susceptibility [19,20,21,22,23,24]. In Brazil, the first autochthonous cases of CHIKV were reported in the municipality of Oiapoque (AP) and in Feira de Santana (BA) in September 2014 [25]. The virus has established itself and, in 2018, a total of 87,687 suspected cases of chikungunya (incidence of 42.1 cases/100,000 inhabitants) and 39 deaths were reported. During this period, the Southeast region had the highest incidence of suspected cases in relation to the country (52,966 cases; 60.4%) and the state of Rio de Janeiro (RJ) had the highest reports. The most affected areas included the municipalities of Rio de Janeiro (10,062 suspected cases; incidence of 150.4/100,000 inhabitants) and Campos dos Goytacazes (7486 suspected cases; incidence of 1487 cases/100,000 inhabitants), in the north of the state [26].

The simultaneous circulation of CHIKV, dengue virus (DENV), Zika virus (ZIKV), and other arboviruses of medical importance, represents a serious public health problem for Brazil, since the overlapping of clinical signs and unavailability of tests make differential diagnosis extremely difficult for health professionals, as well as highlight the need for active and efficient epidemiological surveillance [27,28,29,30]. Here, we aimed to investigate the suspected cases of CHIKV, focusing on differential serological and molecular diagnosis for CHIKV, DENV and ZIKV in the municipalities of Campos dos Goytacazes/RJ and Rio de Janeiro/RJ during an arbovirus outbreak that occurred in 2018.

## 2. Results

### 2.1. Clinical Diagnosis of Chikungunya

**Figure 1:**
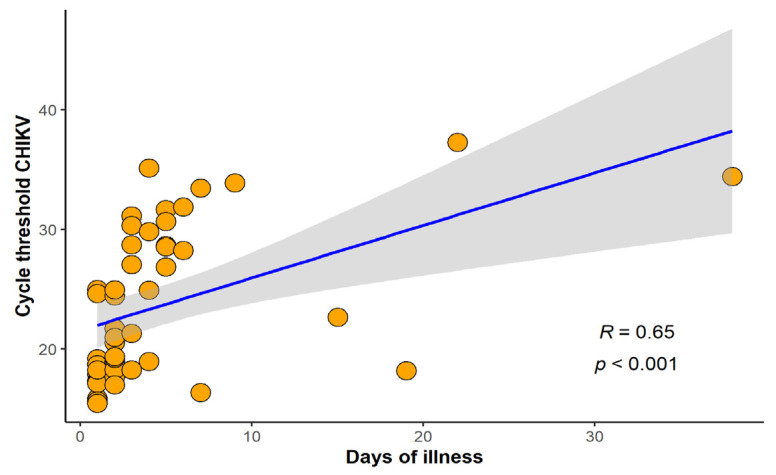
Correlation between cycle threshold chikungunya virus (CHIKV) and number of days of illness. Spearman’s rank correlation coefficient (R) and p-value were calculated between variables (*n* = 48).

In this study, 262 cases of febrile illness compatible with arboviral infection were selected for differential laboratorial diagnosis, at Rio-Laranjeiras Hospital (RJ, *n* = 33) and at Plantadores de Cana Hospital (Campos dos Goytacazes, *n* = 229). Twenty cases were excluded due to lack of information, and therefore, 242 were further investigated. All 242 cases were tested for anti-CHIKV IgM and 63.2% (153/242) were positive, whilst anti-CHIKV IgG was investigated in 164 and was detected in 35.4% (58/164) of the cases. RT-qPCR was performed in 112 cases, and CHIKV infection was confirmed in 42.9% (48/112) of them. The Ct values of patients positive for chikungunya by qRT-PCR ranged from 15.46 to 37.29, with acute cases reaching lower values. The majority of chikungunya cases detected in RT-qPCR were up to 10 days of illness but we were able to detect CHIKV RNA in cases up to 38 days of illness. A positive correlation between days of disease and Ct (r = 0.65, *p* < 0.001) was found (Figure 1).

Overall, among the 242 cases, 73.6% (178/242) were chikungunya RT-qPCR and/or IgM positive and, therefore, considered chikungunya positive cases as recommended [31]. Most cases were in the acute phase (≤14 days of illness), representing 82.6% (147/178), while 17.4% (31/178) were subacute (Table 1).

### 2.2. Baseline Characteristics of Chikungunya Cases

In the chikungunya confirmed cases, 64.0% were female (114/178), 36.0% (64/178) were male. The median age was 44 years old. There were 12 children (≤15 years old), 132 adults (16 to 59 years old) and 34 elderlies (≥60 years old). The acute cases had a lower median age than subacute, being 42 and 51 years, respectively. The most frequently affected age group was adults between 46 to 59 years old (27%) and children were least common (7%), along with the elderly (19%) (Table 1). 

At least one comorbidity was reported by 24% of the patients but no statistical difference was found according to the phase of disease. The most common comorbidity was hypertension, diagnosed in 14% of chikungunya positive cases, most of them acute. Diabetes (4%) and sinusitis (4%) were the second most frequent. Others such as heart disease, tabagism, bronchial asthma, hypothyroidism, allergy, chondromalacia, fibromyalgia, rheumatoid arthritis, glaucoma, Chron’s disease and Alzheimer’s disease were also reported (Table 1).

### 2.3. Clinical Manifestations of Chikungunya Cases

As expected, the most common clinical manifestation reported after fever was polyarthalgy, affecting 89% of patients, acute and subacute. When arthralgia localization was reported, hand/wrist and feet/ankle were more commonly affected (33% and 26%, respectively). Arthralgia in the knees, shoulders and elbows were also reported. Other frequently identified signs and symptoms were: myalgia (69%), headache (62%), exanthema (53%), pruritus (46%), and prostration (46%). We did not find statistically significant differences in the signs and symptoms identified between the acute and subacute phase (Table 1).

### 2.4. Flaviviruses Differential Diagnosis

**Figure 2:**
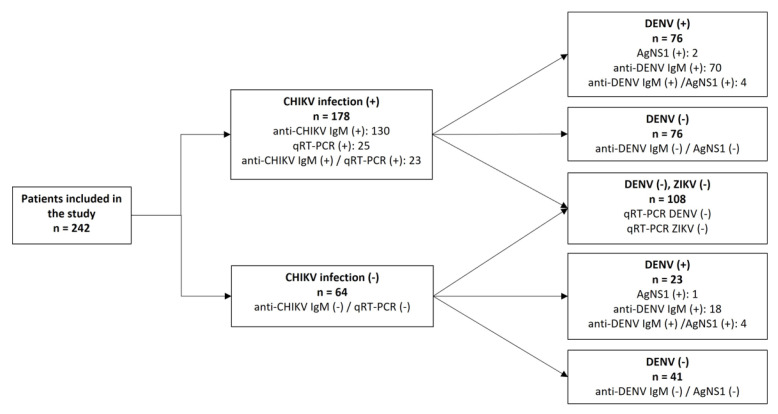
Flowchart summarizing the diagnosis performed in the 242 samples analyzed in this study. CHIKV: Chikungunya virus; DENV: Dengue virus; ZIKV: Zika virus.

Aiming to perform the differential diagnosis with other urban arboviruses co-circulating in RJ, we simultaneously investigated serological evidence of DENV infection using NS1 antigen and anti-DENV IgM ELISA in all 242 cases. Only 4.5% (11/242) were positive for DENV NS1 antigen, and 39.7% (96/242) were anti-DENV IgM positive. Due to the high incidence of chikungunya cases in an endemic scenario to dengue and Zika, we randomly selected 108 cases to investigate the presence of DENV and ZIKV’s RNA through RT-qPCR and they were all negative (Figure 2). However, because the cases were not serologically tested to Zika antibodies due to notorious cross-reactivity on the antibody responses generated in response to flavivirus infections, we are unable to exclude a recent or past exposure to ZIKV, characterized by the detection of specific IgM or IgG. 

Overall, we identified 23 (9.5%) exclusive cases of recent dengue (IgM and/or NS1 positive), 102 (42.1%) were considered as chikungunya exclusive cases by the detection of specific antibody and/or viral genome, and in 76 (31.4%) patients, the co-detection of chikungunya and recent dengue exposure (here, named chikungunya/dengue) was reported. A total of 41 patients were negative after all diagnostic testing. No significant differences were found in the CHIKV cycle threshold values between the chikungunya cases and the chikungunya/dengue cases.

In order to find differences in clinical manifestations presented by the recent dengue cases, we analyzed symptoms reported by three different groups: chikungunya cases (anti-CHIKV IgM and/or RT-qPCR positive), recent dengue (anti-DENV IgM and/or NS1 positive), and chikungunya/dengue cases (simultaneously positive by any laboratorial method). The median age of the groups ranged between 43 and 44 years old. We found a higher frequency of women with a diagnosis of chikungunya, while a higher proportion of men with recent dengue was observed, but differences were not statistically significant (Table 2). When comparing the presence of comorbidities between the three groups, we found that patients with dengue had a lower frequency of comorbidity in relation to chikungunya monoinfection and chikungunya/dengue cases (*p* = 0.004). Chikungunya positive cases had a significantly higher frequency of hypertension (*p* = 0.013) and sinusitis (*p* = 0.026) compared to dengue patients and to chikungunya/dengue cases. Patients with a negative test for dengue, Zika and chikungunya had a significantly higher frequency of cough (*p* = 0.046), probably related to respiratory infections. Fever, polyarthralgia, myalgia, exanthema and headache were the most common signs and symptoms on all groups analyzed. Nonetheless, chikungunya and chikungunya/dengue cases, reported more frequently the location of arthralgia (74% and 58%, respectively) than recent dengue cases (29%). Prostration was more frequent in chikungunya patients, although this difference was not statistically significant. Conjunctival hyperemia was more frequently reported in chikungunya/dengue cases. Arthritis and edema of the limbs were more frequently observed in chikungunya cases. Moreover, those patients had a significantly higher frequency of arthritis (*p* = 0.023) and lower limb swelling (*p* < 0.001), compared to the other two groups (Table 2).

### 2.5. Genotype Characterization of Representative CHIKV Strains Circulating in Rio de Janeiro in 2018 

**Figure 3:**
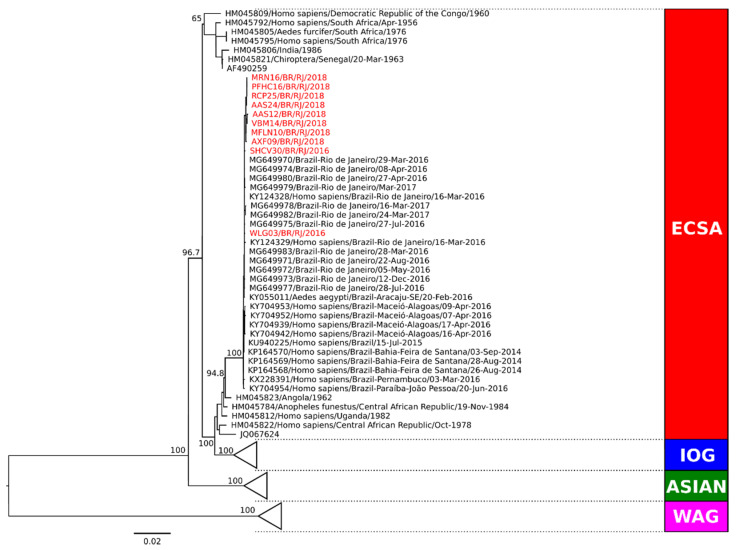
Phylogenetic analysis of the complete coding sequence of representative CHIKV strains from Campos dos Goytacazes and Rio de Janeiro during 2018. Maximum likelihood (MV) method, bootstrap of 1000 replications. The analyzed CHIKV sequences are represented in red color and designated as follows: strain name/country/city/year). Sequences representing the ECSA, Indian Ocean, Asian and West African genotypes obtained from GenBank were used as reference. ECSA: East-Central-South African genotype; IOG: Indian Ocean lineage/genotype; WAG: West African genotype.

Phylogenetic analysis of the complete genome coding region was performed in representative strains from Campos dos Goytacazes (*n* = 5) and from Rio de Janeiro (*n* = 3) for genotype characterization. Two additional CHIKV strains from RJ from 2016 were also included for surveillance and comparison purposes. Sequences representing the Asian, ECSA, and West African genotypes obtained from GenBank were used as reference. Our comparative analysis showed that all CHIKV strains were characterized as belonging to the ECSA genotype (Figure 3). Molecular characterization was also performed (data not shown) and no amino acid differences were observed in RJ strains from 2018. The analysis of the E1 protein region did not demonstrate the A226V mutation, revealing that the amino acid alanine was present in the E226 position of all strains. However, we did find a change at the position E305 in strains from 2016, where a threonine was replaced by an alanine.

## 3. Discussion

The present study investigated suspected cases of arboviral infection during an epidemic in two different cities in state RJ, Brazil in 2018, focusing in differential diagnosis of CHIKV, DENV and ZIKV infections, all of them circulating in Brazilian territory at the time [26]. Due to the epidemiological scenario with the co-circulation of arboviruses in the Southeast region, surveillance studies such as the one presented here play a key role in understanding the impact of those viruses in Brazil.

DENV has been circulating in Brazilian territory since 1986 [32], the first autochthonous cases of CHIKV were reported in Brazil in 2014 [25], and ZIKV emerged in 2015 [33], resulting in the co-circulation and triple epidemics in some states of the country [7,34,35]. As the disease caused by those viruses share similar signs and symptoms, clinical differential diagnosis is challenging for health. Moreover, laboratorial diagnosis can be troublesome, specially between ZIKV and DENV, as they belong to same family [27,36]. Here, in spite of a triple arboviral epidemic in the State, 73.6% (178/242) of the febrile illness investigated were CHIKV positive. In those cases, 42.1% (102/242) were positive only in chikungunya tests. In 9.5% (23/242) of the cases analyzed, patients were dengue positive to anti-DENV IgM and/or NS1 antigen detection. Moreover, 42.7% (76/178) of the chikungunya cases also presented recent exposure to DENV, characterized by anti-DENV IgM and/or NS1 detection and reflecting a scenario of those viruses’ co-circulation. Despite that, no DENV or ZIKV viral genomes were detected by molecular diagnosis. The DENV NS1 protein is a reliable marker for early diagnosis of dengue because it is highly secreted during viral replication in infected individuals [37,38] and it does not cross react with ZIKV infections [39]. Although it can be detected at the same time as the viral RNA, the protein may be found circulating up to 9–14 days after the onset of the disease [40], thus when the virus is no longer circulating. Likewise, anti- DENV IgM can be elicited as early as 3 to 5 days after the disease onset, and can remain detectable for up to 90 days [41]. Therefore, in this study, dengue positive cases for anti- DENV IgM and NS1 antigen, and with a negative result for viral genome detection, were considered as a recent exposure to DENV and cases serologically positive for both dengue and chikungunya, as co-detections and not co-infections.

A total of 87,687 chikungunya cases were reported in Brazil during 2018, and the state of RJ alone reported 39,725 cases. In that same year, dengue cases were 3-fold higher than that reported for chikungunya in the country, but in RJ, only 14,992 cases were reported. A low circulation of ZIKV in the state was also observed, where 2349 cases were reported [26] and, therefore, an intense circulation of CHIKV was characterized in the state. DENV and CHIKV coinfections are frequently reported by endemic countries [42]. In this study, the percentage of chikungunya/dengue co-detections (42.7%) was higher than that found in previous years in Brazil [35,43], although a meta-analysis study found that the magnitude of coinfections may range from 0 to 32% [44]. Despite that, the high proportion found here was similar to that observed in other countries in the Americas region [45,46]. As the DENV genome was not detected in the cases analyzed, the high number of both anti-DENV IgM and anti-CHIKV positive cases may potentially be related to a potential cross-reactivity of the CHIKV IgM ELISA test with anti-DENV IgM positive cases reported previously in the country [47]. Therefore, the availability of molecular tests for a reliable differential diagnosis is crucial for patient’s management, particularly in areas with high circulation of both arboviruses [48,49].

Although being considered a triple epidemic, we found a higher number of chikungunya cases (102), followed by dengue (23), and no cases of Zika. These results are in agreement with the information that the arbovirus’ space–time distribution is different during an epidemic [50,51]. During the peak of the triple epidemic in 2015–2016, the spatial analysis and the incidence of dengue, Zika and chikungunya in the city of RJ showed that only 25% of the studied areas had a high incidence of the three arboviruses [51] and that they have formed disease transmission clusters, with only 31% of them transmitting all three arboviruses simultaneously [50]. Some explanations for this phenomenon are that there is competition between viruses, especially between CHIKV and ZIKV, both in nature and in the laboratory [52].

CHIKV infection was confirmed in 42.9% of the cases tested by molecular diagnosis, including convalescent cases of over 14 days after the onset of symptoms, and interestingly, one of those cases was collected after 38 days of illness. In humans, the persistence of CHIKV RNA in perivascular macrophages from the synovial fluid of a chronic patient was demonstrated for up to 18 months after infection, and this can be explained by the exhaustion of T cells due to the strong immune response during the acute phase, as a consequence of this viral persistence [53]. Further studies with humans will be needed to clarify how and for how long the CHIKV viral RNA persists, as well as its relationship with the patient’s immune system and the clinical course of chronic arthralgia [54]. To date, viral persistence has been shown to be directly associated with immune ineffectiveness and efficient viral escape [53].

It has been shown that CHIKV/DENV coinfections and high DENV viral loads contributed to severe manifestations among infected patients [55]; however, in this study, we were unable to access dengue viremia. Moreover, by presence of anti-DENV IgM in the cases analyzed here, it is more likely that DENV infection occurred prior to the CHIKV one.

Chikungunya shares signs and symptoms with dengue and Zika, and can be misdiagnosed in areas where these arboviruses circulate [56]. In this study, polyarthralgia, fever, myalgia, headache, exanthema and prostration were more frequently reported, all commonly related to DENV and CHIKV monoinfections and CHIKV/DENV coinfections [57,58,59,60,61,62]. Even so, chikungunya cases had a significantly higher frequency of arthritis (*p* = 0.023) and lower limb swelling (*p* < 0.001) compared to dengue and chikungunya/dengue cases. The higher frequency of joint involvement in CHIKV monoinfections compared to coinfections has already been evidenced in a previous study [62].

Conjunctival hyperemia, a characteristic sign of chikungunya and Zika [63], was found more frequently in chikungunya and chikungunya/dengue cases. Overall, polyarthralgia was most commonly reported in both acute and subacute phases of the chikungunya cases. Fever, headache and retro-orbital pain were frequent in the acute phase, while exanthema and itching were more frequent in the subacute ones. Skin involvement is frequently reported during chikungunya fever [64], and may be characterized by rashes, ulcers, dermatoses, erythema [57].

Twenty-three percent of patients reported a comorbidity, and among them, patients with dengue had a lower frequency of comorbidity in relation to chikungunya monoinfection and chikungunya/dengue cases (*p* = 0.004). The presence of comorbidities such as hypertension and diabetes mellitus may indicate predisposition to severe chikungunya [65,66]. However, we were not able to do a follow up of these patients.

According to Fabri et al. (2020), since 2015, two independent CHIKV-ECSA genotype introductions occurred in RJ, both from Brazil’s northeastern region [67]. The first report and characterization of CHIKV-ECSA in RJ occurred in 2016 [68]. Here, we confirmed that the representative CHIKV strains analyzed and circulating in the cities of Campos do Goytacazes and RJ in 2018 also belonged to the ECSA genotype and did not demonstrate the A226V mutation on the E1 gene, known to increase the fitness on the *Ae. albopictus* vector and not found previously on strains from RJ [35]. Molecular characterization showed the absence of significant mutations in these strains, but when comparing the 2018 strains with representative ones, collected in RJ in 2016, the T305A mutation was found in the E protein region from the latter. To the best of our knowledge, it is the first time this mutation has been described. Changes in nearby regions have also been reported, such as E1:I317V and V322A identified in India [69,70]. As the possibility of mutations in the E protein favoring viral transmission is well known, it is important to develop new studies with a higher number of samples and to investigate the relevance of these mutations.

Although DENV, ZIKV and CHIKV are still currently co-circulating in RJ and Brazil, a change in the epidemiological profile has been observed after 2017, with co-infections no longer identified as previously [7,71]. Despite that, due to the ongoing co-circulation of those viruses, the challenge for the clinical differential diagnosis still remains. In that scenario, the laboratorial differential diagnosis based on molecular approaches is crucial.

## 4. Conclusions

Here, we identified that 73.6% of patients with fever and joint pain in our study group were chikungunya cases and the incidence of coinfection CHIKV/DENV (31.4%) was higher than in previous studies. Arthritis and lower limb swelling were significantly more frequent in patients with CHIKV monoinfections compared to patients exposed to DENV or to both viruses. The laboratory diagnosis for arboviruses is of fundamental importance for differentiating cases during multiple epidemics, which will directly impact the correct clinical management of the patient. We did not find any Zika cases and most of the chikungunya and dengue cases were identified by serological methods. Phylogenetic analysis showed that ECSA was the circulating genotype and, when compared to strains from two years before, a mutation (T305A) exclusive to the 2016 strains was observed. The circulation of multiple arboviruses, as occurs in Brazil, represents a public health challenge, and it is necessary to strengthen the differential diagnosis by molecular methods, aiming not only for better medical assistance, but also the investigation of the epidemics’ dynamics for a better management of health politics.

## 5. Materials and Methods

### 5.1. Study Sites and Sample Collection

**Figure 4:**
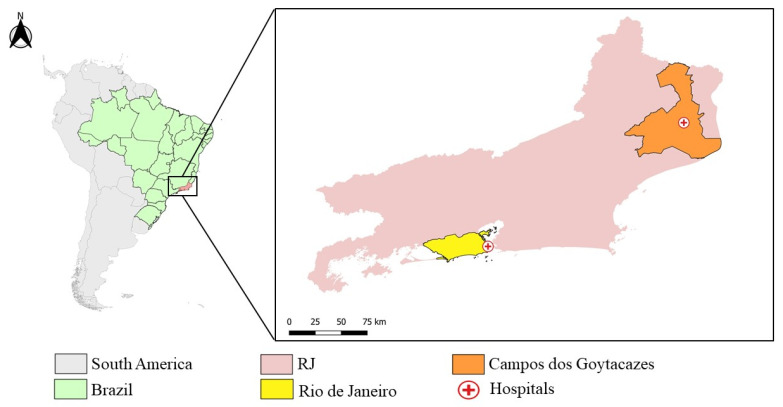
Map of Rio de Janeiro, highlighting the city Rio de Janeiro (yellow) and Campos dos Goytacazes (Orange) and the Hospital Plantadores de Cana and Hospital Rio Laranjeiras’ location (red cross). (The map was prepared in QGIS software. Maps from IBGE, Brazillian base map: https://www.ibge.gov.br/geociencias/organizacao-do-territorio/malhas-territoriais.html, accessed on 20 December 2021).

RJ is the third most populated state in Brazil, with 17,463,349 million inhabitants and 43,750,426 km² of territory, located in the Brazilian southeast region [72]. The state’s capital, the city of RJ, is located in the south of the state, and has an estimated population of 6,775,561 people and 1,200,329 km² [73]. Campos dos Goytacazes, located in the north of the state (Figure 4) has a bigger area (4,032,487 km²), but a smaller population of 514,643 people [74]. 

In this study, samples of serum, plasma, and whole blood from suspected cases of CHIKV and/or DENV and/or ZIKV from patients that experienced a febrile illness accompanied by intense polyarthralgia and attended at two distinct hospitals in RJ, were collected during a cross-sectional and observational study performed by the Viral Immunology Laboratory (LIV, IOC/FIOCRUZ). A total of 262 samples were collected, 33 at Rio-Laranjeiras Hospital (RJ) and 229 at Plantadores de Cana Hospital (Campos dos Goytacazes) (Figure 4).

The inclusion criteria included patients of any age group and gender, who experienced a febrile illness accompanied by intense polyarthralgia, according to the Ministry of Health, 2014 [31], and exclusion criteria included patients that did not agree to participate in the study or who were suspected of other infections. The investigations in Rio de Janeiro were performed from March to April 2018, and in Campos dos Goytacazes, from June to September, 2018. During investigations, an infectious disease physician collected data on demographics and signs and symptoms using a structured questionnaire. Plasma samples were submitted to a serological and/or molecular laboratory diagnosis for confirmation or exclusion of DENV, ZIKV and CHIKV infections. Of these, 143/262 (54.58%) were submitted to serology only and 119/262 (45.41%) were tested simultaneously by serological and molecular methodologies.

### 5.2. Serological Diagnosis of Chikungunya and Dengue

Serological diagnosis was performed using commercial kits, according to the manufacturer’s protocol. For dengue, the Panbio dengue IgM Capture ELISA kit (Alere™, Brisbane, Australia) and ELISA Platelia™ Dengue NS1 Ag-ELISA (BioRad Laboratories, Hercules, CA, USA) were used. Anti-CHIKV antibodies were detected using Anti-CHIKV ELISA IgM and IgG kit (Euroimmun, Lubeck, Germany), respectively. We did not perform Zika serological tests due to its cross reactivity with anti-DENV antibodies.

### 5.3. Molecular Diagnosis

For arboviruses molecular detection, total RNA was extracted from the plasma of suspected cases using the QIAamp Viral RNA Mini kit (Qiagen, Hilden, Germany), following the manufacturer’s protocol. The viral RNA was stored at −70 °C for subsequent molecular diagnosis. The real-time reverse transcriptase polymerase chain reaction (RT-qPCR) for CHIKV detection was performed according to Lanciotti et al. [75]. For DENV detection and serotyping, the conventional semi-nested RT-PCR described by Lanciotti et al. [76] and the RT-qPCR described by Johnson et al. [77] were used. For ZIKV detection, the RT-qPCR was performed according to Lanciotti et al. [78].

### 5.4. Chikungunya Virus Genotyping

Representative chikungunya positive cases by qRT-PCR (*n* = 8) were randomly selected for genotyping by sequencing the complete coding region of the virus genome. Sets of primers were designed to amplify overlapping fragments of the CHIKV complete genome coding region (Table 3) and were purified using the PCR Purification Kit or Gel Extraction Kit (Qiagen, Inc., Frankfurt, Germany) and sequenced in both directions using the BigDye Terminator Cycle Sequencing Ready Reaction version 3.1 kit (Applied Biosystems^®^, Foster City, CA, USA). The thermocycling conditions consisted of 40 cycles of denaturation (94 °C/10 s), annealing (50 °C/5 s) and extension (60 °C/4 min). Sequencing was performed on an ABI 3730 DNA Analyzer, Applied Biosystems^®^, CA, USA. The phylogenetic analysis was performed using the nucleotide sequences of different representative strains of the CHIKV available in the National Center for Biotechnology Information database (http://www.ncbi.nlm.nih.gov, accessed on 20 December 2021), using the coding regions of the structural and non-structural proteins, as well as intergenic regions. The untranslated region (UTRs) 5’ and 3’ were excluded from the analysis. The dataset generated, along with the sequences analyzed here, were submitted to multiple sequence alignment (MSA), using the Mafft v.7 software [79], and edited, when necessary, using the Geneious v.9.1.8 software (https://www.geneious.com/, accessed on 20 December 2021). The aligned dataset was submitted to identify the best nucleotide substitution model, then the construction of the phylogenetic trees was carried out using the maximum likelihood (MV) method [80], using the IQ-TREE v.1.6.12 software [81], with a bootstrap of 1000 replications to provide greater reliability to the grouping values [82]. The phylogeny visualization was performed by the FigTree v.1.4.4 software (https://github.com/rambaut/figtree/releases/tag/v1.4.4, accessed on 20 December 2021). For the dataset used, we chose not to use a root sequence; for this reason, the midpoint rooting method was used, a tool available in the phylogeny visualization program.

### 5.5. Statistical Analysis

The Shapiro–Wilk test was used to assess the normality of the measured data; discrete variables were presented as percentages and continuous variables were presented as means with standard deviations (SDs) or as medians with interquartile ranges (IQRs), as appropriate. The Mann–Whitney U test, chi-square test and Fisher’s exact test were used to compare differences between groups, as appropriate. All statistical analyses were conducted in STATA version 15.0 (StataCorp, College Station, TX, USA); *p* values < 0.05 were considered statistically significant.

## Figures and Tables

**Table 1:** Demographic and clinical characteristics of Chikungunya cases according to the phase of the disease (*n* = 178), investigated during a triple arboviral epidemic in RJ, 2018.

	Total *n* = 178		Acute *n* = 147		Subacute *n* = 31		*p*
**Age (years old)**							
median, IQR	44	(28–56)	42	(26–56)	51	(33–62)	0.063
Age groups							
≤15 years	12	7%	11	7%	1	3%	
16–30 years	42	24%	38	26%	4	13%	
31–45 years	42	24%	34	23%	8	26%	
46–59 years	48	27%	39	27%	9	29%	
≥60 years	34	19%	25	17%	9	29%	
**Sex**							
Male	64	36%	56	38%	8	26%	0.195
Female	114	64%	91	62%	23	74%	
**Comorbidity**							
Overall	42	24%	32	22%	10	32%	0.211
**Stratified**							
Hypertension	25	14%	18	12%	7	23%	0.155
Diabetes	8	4%	5	3%	3	10%	0.145
Sinusitis	7	4%	4	3%	3	10%	0.103
Rhinitis	4	2%	3	2%	1	3%	0.538
Asthma	2	1%	1	1%	1	3%	0.319
Heart disease	2	1%	2	1%	0	0%	1.000
Others	5	3%	4	3%	1	3%	1.000
**Signs and symptoms**							
Fever	178	100%	147	100%	31	100%	1.000
Polyarthralgia	158	89%	131	89%	27	87%	0.746
Location not reported	52	33%	43	33%	9	33%	
Location reported *	106	67%	88	67%	18	67%	
Hand/wrist	35	33%	29	33%	6	33%	
Foot/ankle	28	26%	22	25%	6	33%	
knee	21	20%	19	22%	2	11%	
shoulder	14	13%	12	14%	2	11%	
elbow	8	8%	6	7%	2	11%	
Myalgia	123	69%	101	69%	22	71%	0.805
Headache	111	62%	93	63%	18	58%	0.587
Exanthema	95	53%	78	53%	17	55%	0.857
Prostration	81	46%	67	46%	14	45%	0.966
Pruritus	82	46%	63	43%	19	61%	0.061
Nausea	67	38%	57	39%	10	32%	0.496
Retro-orbital pain	66	37%	57	39%	9	29%	0.307
Hyporexia	58	33%	50	34%	8	26%	0.376
Lower limb swelling	57	32%	46	31%	11	35%	0.649
Lower back pain	49	28%	42	29%	7	23%	0.497
Vomiting	34	19%	24	16%	10	32%	0.040
Diarrhea	31	17%	23	16%	8	26%	0.175
Asthenia	27	15%	23	16%	4	13%	1.000
Conjunctival hyperemia	26	15%	23	16%	3	10%	0.577
Dizziness	27	15%	23	16%	4	13%	1.000
Abdominal pain	24	13%	21	14%	3	10%	0.772
Arthritis	14	8%	11	7%	3	10%	0.714
Cough	14	8%	14	10%	0	0%	0.134
Paresthesia	12	7%	10	7%	2	6%	1.000
Chills	11	6%	11	7%	0	0%	0.216
**Co-detection**							
Chikungunya/dengue	76	43%	62	42%	14	45%	0.760

(*) the relative percentage was calculated for the locations reported.

**Table 2:** Demographic and clinical characteristics of chikungunya, dengue and chikungya/dengue cases investigated during a triple arboviral epidemic in RJ, 2018.

	Chikungunya Cases *n* = 102		Recent Dengue Cases *n* = 23		Chikungunya/Dengue Cases *n* = 76		*p*
**Age (years)**							
median, IQR	43	(28–59)	44	(38–56)	44	(28–56)	0.833
**Sex**							
Male	36	35%	14	61%	28	37%	0.068
Female	66	65%	9	39%	48	63%	
**Comorbidity**							
**Overall**	32	31%	2	9%	10	13%	0.004
**Stratified**							
Hypertension	20	20%	1	4%	5	7%	0.013
Diabetes	5	5%	1	4%	3	4%	1.000
Sinusitis	7	7%	0	0%	0	0%	0.026
Rhinitis	4	4%	0	0%	0	0%	0.233
Asthma	1	1%	0	0%	1	1%	1.000
Heart disease	2	2%	0	0%	0	0%	0.614
Others	4	4%	0	0%	1	1%	0.543
**Signs and symptoms**							
Fever	102	100%	23	100%	76	100%	1.000
Polyarthralgia	92	90%	17	74%	66	87%	0.109
Location not reported	24	26%	12	71%	28	42%	
Location reported *	68	74%	5	29%	38	58%	
Hand/wrist	23	34%	2	40%	12	32%	
Foot/ankle	17	25%	1	20%	11	29%	
Knee	13	19%	0	0%	8	21%	
Shoulder	8	12%	0	0%	6	16%	
Elbow	7	10%	2	40%	1	3%	
Myalgia	72	71%	15	65%	51	67%	0.824
Exanthema	60	59%	12	52%	35	46%	0.239
Headache	66	65%	13	57%	45	46%	0.654
Prostration	51	50%	7	30%	30	39%	0.147
Pruritus	54	53%	9	39%	28	37%	0.084
Nausea	41	40%	7	30%	26	34%	0.570
Retro-orbital pain	45	44%	8	35%	21	28%	0.077
Hyporexia	37	36%	3	13%	21	28%	0.074
Lower limb swelling	40	39%	0	0%	17	22%	<0.001
Lower back pain	33	32%	6	26%	16	21%	0.244
Vomiting	22	22%	6	26%	12	16%	0.464
Diarrhea	15	15%	7	30%	16	21%	0.183
Asthenia	17	17%	2	9%	10	13%	0.570
Conjunctival hyperemia	15	15%	1	4%	11	14%	0.397
Dizziness	20	20%	3	13%	7	9%	0.151
Abdominal pain	17	17%	0	0%	7	9%	0.054
Arthritis	12	12%	0	0%	2	3%	0.023
Cough	11	11%	3	13%	3	4%	0.189
Paresthesia	9	9%	0	0%	3	4%	0.238
Chills	7	7%	0	0%	4	5%	0.559

(*) the relative percentage was calculated for the locations reported.

**Table 3:** Oligonucleotide primers used for the amplification of the complete genome coding region of the CHIKV strains.

Primer Identification	Primer Sequence (5′-3′)	* Position in the Genome
CHIK 1A	ACT GCT CTA CTC TGC AAA GC	39_F
CHIK 1B	CTC CGG CGT GAC TTC TGT A	1136_R
CHIK 2A	CCG TGT GCT GTT CTC AGT AG	_788_F
CHIK 2B	GTT CTG CTT CTC GTT CTT CC	1590_R
CHIK 3A	AGG AGT GCC GGA AAG ACA TG	1264_F
CHIK 3B	CCT GCA GCT TCT TCC TTC	2128_R
CHIK 4A	TGG TAC TTT CCC CGC AGA C	1756_F
CHIK 4B	TCA CAG GCA GTG TAC ACC	2634_R
CHIK 5A	GGC AAG ACC TGG TGA CTA GC	2287_F
CHIK 5B	ATA GGG ACC AAG CTC TTA GC	_3139_R
CHIK 6A	GTG CTT CAG AGG GTG GGT TA	2756_F
CHIK 6B	GTG ACT CTC TTA GTA GGC AG	3637_R
CHIK 7A	CCT GAA TGA AAT ATG CAC GCG C	3233_F
CHIK 7B	TTC TTC GCG ATG TCC ATG C	4117_R
CHIK 8A	ACG CAA TGA AAC TGC AAA TG	3784_F
CHIK 8B	CGT GGT GCT GTA TCC TTT TC	4655_R
CHIK 9A	CCT ATC GAG AAG TCG CAA AG	4339_F
CHIK 9B	ATT ACC CAG TCA GAC ACG G	5257_R
CHIK 10A	GAG CAA GTC TGC CTA TAT GC	4758_F
CHIK 10B	ACG TGG ACC AGT CGC TAT C	5622_R
CHIK 11A	ACT GGG TAA TGA GCA CCG TAC	5248_F
CHIK 11B	TGA CGG ATT GAA TGT CGC TC	6167_R
CHIK 12A	ACG AGG AGA AGT GTT ACC CAC	5758_F
CHIK 12B	GCC TGT ATA ACC TGC ACC	6607_R
CHIK 13A	GCA ACG TCA CAC AGA TGA GG	6286_F
CHIK 13B	CCA TCA ATT CAT CGG AGA CG	7104_R
CHIK 14A	AGC CGC ACA CTT TAA GCC AG	6737_F
CHIK 14B	AGG CTG GTA CCT CCT ATT G	7615_R
CHIK 15A	ATC AGA TGG CAA CGA ACA GG	7329_F
CHIK 15B	GTG GTG CCA GTT GTA GTA C	8140_R
CHIK 16A	CGG AAG AAT AAG AAG CAA AAG C	7760_F
CHIK 16B	AGT GCC CTT CTC CAC AGT C	8621_R
CHIK 17A	AAT GAA GGA GCC CGT ACA GC	8264_F
CHIK 17B	TTG CCG GAC TGT TGT GAC	9102_R
CHIK 18A	ACC GTG CAC GAT TAC TGG AAC	8809_F
CHIK 18B	CAG AAT TAT CTC ATG CGG GTG G	9613_R
CHIK 19A	TGC AGG GTG CCT AAA GCA AG	9338_F
CHIK 19B	GTA ATC AAG CGA TAG CGT TGG	10132_R
CHIK 20A	TAC CGT CCC TTT CCT GCT TA	9760_F
CHIK 20B	AAT TGT CCT GGT CTT CCT GC	10593_R
CHIK 21A	GAA GTC CGA ATC ATG CAA AAC	10321_F
CHIK 21B	GTG TAC TTG TGT AGA ACA GAC	11119_R
CHIK 22A	AGC AAC AAA CCC GGT AAG AG	10777_F
CHIK 22B	TAG TTG TCA AGT TAG TGC CTG C	11325_R
CHIK 23A	ATG GGT GCA GAA GAT CAC G	11218_F
CHIK 23B ECSA	GTA TAG CCC TTT GAA CTA CTT C	11613_R
CHIK 23B ASIAN	GCT ATA TAT GGT GTG TCT CTT AGG	11522_R

* According to CHIKV strain Genbank accession number KP164570.1. F: Forward; R: Reverse.

## Data Availability

Detailed data on the patients’ demographic, as well clinical and laboratorial data presented in this study, are available on request from the corresponding author due to the patients’ privacy. CHIKV genome sequences data were submitted to GenBank.
